# Deep Vibro-Tactile Perception for Simultaneous Texture Identification, Slip Detection, and Speed Estimation

**DOI:** 10.3390/s20154121

**Published:** 2020-07-25

**Authors:** Yerkebulan Massalim, Zhanat Kappassov, Huseyin Atakan Varol

**Affiliations:** Department of Robotics and Mechatronics, Nazarbayev University, Nur-Sultan 010000, Kazakhstan; yerkebulan.massalim@nu.edu.kz (Y.M.); ahvarol@nu.edu.kz (H.A.V.)

**Keywords:** tactile sensing, slip detection, texture identification, deep learning, convolutional neural networks, long short-term memory, accelerometers

## Abstract

Autonomous dexterous manipulation relies on the ability to recognize an object and detect its slippage. Dynamic tactile signals are important for object recognition and slip detection. An object can be identified based on the acquired signals generated at contact points during tactile interaction. The use of vibrotactile sensors can increase the accuracy of texture recognition and preempt the slippage of a grasped object. In this work, we present a Deep Learning (DL) based method for the simultaneous texture recognition and slip detection. The method detects non-slip and slip events, the velocity, and discriminate textures—all within 17 ms. We evaluate the method for three objects grasped using an industrial gripper with accelerometers installed on its fingertips. A comparative analysis of convolutional neural networks (CNNs), feed-forward neural networks, and long short-term memory networks confirmed that deep CNNs have a higher generalization accuracy. We also evaluated the performance of the highest accuracy method for different signal bandwidths, which showed that a bandwidth of 125 Hz is enough to classify textures with 80% accuracy.

## 1. Introduction

Friction is undoubtedly important to humans. Without it, for instance, we could not manipulate an object or even walk. Friction has attracted the attention of scientists since the era of Leonardo da Vinci, who pioneered the investigation of friction and slippage [1]. Frictional resistance between an object and our hand lets us grasp an object when we apply sufficient force at the points of contact [2]. This force prevents the object from slipping and, therefore, from falling. Even though we may grasp objects—when volumetric boundaries of an object fit into the hand—with the form-closure [3], the true dexterity emerges when we grasp and manipulate objects with the force-closure [2]. We finely control both the fingertip motions [4] and the forces at the contact points with a grasped object to prevent its slippage [5]. Thanks to the sense of touch in our fingers [6,7,8], these forces can increase almost immediately if the object starts to slip since our hands are able to detect the friction-induced vibrations (FIVs). Indeed, the dexterity of manipulation is not affected by the visual acuity of a person. On the other hand, in the absence of the sense of touch—e.g., through anesthetizing the fingertips as was demonstrated in the experiments on lightning matches [9] –, the dexterity of the manipulation attenuates dramatically.

The essential goal during object manipulation and grasping is to avoid slippage. Its success depends on both detecting the slippage and identifying the surface of the object. Earlier studies focused on the detection of either the slippage or the surface texture [10]. In contrast, we aim at distinguishing contact events, such as slip and textures, simultaneously. The contribution of this paper is the method for concurrent slip and texture detection using a robot gripper equipped with a FIV detection system.

We are motivated by the sensory-motor system of the human hand. Edin et al. [11] revealed that coordination of finger-tip forces happens independently when an object is manipulated between two opposite fingers. Specifically, with identical textures at the grip surfaces, the subjects exerted approximately equal forces at the two fingertips; whereas, with different textures at the two grip surfaces, subjects applied different forces. This suggests that texture identification and slip detection happen simultaneously in human hands. Indeed, people parametrically adapt to different surface conditions and use less grip forces on rougher surfaces [12]. Therefore, texture discrimination and slip detection abilities are critical for forming and updating predictions when adapting forces to object surface properties. A time delay between 30 ms and 100 ms to update the grip force was reported [13].

Inspired by this finding, we developed a system consisting of accelerometers installed in robot fingertips (Section 3). Signals from the accelerometers were organized as a dataset (4) and analyzed using deep learning (Section 5) to detect the slip, and classify the surface texture with less time delay (around 17 ms) than humans. The slip occurs when the grasped object moves with respect to the gripper. This slip is discriminated from the sliding motion—the grasped object is static within the gripper, but an external object slides over it. Presumably, this enhancement will bring robot hands one step closer to dexterous manipulation [14,15]. In Section 6, we present the benchmarking of these techniques and we conclude the work with Section 7. The following section (Section 2) overviews the related work.

## 2. Slip And Texture Detection

National Institute of Standards and Technology (NIST) workshop on dexterous manipulation highlighted tactile sensing as an important enabler for autonomous robotics [16]. In this connection, the research on artificial tactile sensing has been flourishing in recent decades [10]. Our approach leverages prior work in slip and texture detection. Some of the existing methods build upon one dimensional (1D) measurements of FIVs, e.g., using accelerometers embedded in robot fingertips [17,18] and in hand prostheses [19]. Others, rather, indirectly use two dimensional (2D) camera images, e.g., optical sensing [20] embedded in a robot end-effector.

### 2.1. Image-Based Methods

These approaches predict the onset of a slip thanks to incipient slip [21]. Research on human tactile sensing elucidated this phenomenon by observing the evolution of slip in the contact region [22]. Accordingly, some investigators developed tactile sensing arrays and treated their output as grayscale images [23], while others used cameras and computer vision algorithms [24]. In both cases, tactile features moving across sa 2D image were tracked.

Capable of capturing a 2D contact pattern at once, the camera- and array-based sensors were used to classify surface textures and objects [25]. For example, a planar pressure profile sensing array—with its output processed by convolutional neural networks (CNNs)—was used to identify object footprints [26]. The volumetric shape of an object was reconstructed from multiple footprints acquired by multiple computerized palpations [27,28]. However, the spatial resolution of these tactile sensing arrays is too low to detect microscale texture features; the sizes of the sensing elements are in the order of mms, whereas texture patterns are of μm range. For this reason, high resolution cameras have been shown as effective optical tactile sensors for texture identification [29]. Even though vision cameras have been used in tactile sensing since 1990s [30,31], a breakthrough was achieved using a transparent elastomer that was coated with golden/silver powder from one side, which acts as a reflective surface, and a vision camera from the opposite [32].

To some extent, the sensing arrays, e.g., with electro-conductive rubber [33], and optical tactile sensors, e.g., Gelsight [34], TacTip [35], share the same drawback—the contact surface tears and wears off, resulting in deteriorated sensing performance.

### 2.2. Force Vector Measuring Methods

Coulomb friction cone estimation is one of the earliest problems discussed in grasping for ensuring stable manipulation. Many of the demonstrated solutions focused on the friction estimation between two interacting surfaces from the observations of the normal and tangential forces. Specifically, the undesirable slippage is avoided by maintaining objects within static frictional safety margins [36]. More precise dynamic friction models, e.g., LuGre friction model, was used to predict slippage [37]. A similar approach was applied for preventing rotational slippage by estimating rotational friction [38].

The friction between a sensor and an object depends on the surface properties of the object, e.g., texture. Sliding over a surface can be accomplished using tactile servoing [39]. It is possible to classify objects based on the time-sequence of friction coefficients after the sliding motion [40]. An analogous approach with more sensors (pressure sensing barometers and electro-conductive electrodes) was presented in [41]. In the latter work, the traction of a surface was selected as one of surface features for the texture classification.

The methods that are based on force sensing require precise knowledge about the surfaces in contact. The vibration-based methods discussed in Section 2.3 are invariant to the surface properties.

### 2.3. Vibration Detection Methods

The methods within this group directly measure vibrations in the fingertip medium. These vibrations are produced by a slip or by dragging the fingertip across a textured surface. In an earlier work, the investigators captured these vibrations with accelerometers to detect the slippage in a hand prosthesis [19]. These vibrations can be recorded using piezoresistive elements [42], capacitive cells [43], barometers with fluid media [41], and recently neuromorphic cameras [44].

The slippage can be detected by observing the high frequency components of a signal [15]. However, this requires some tuning of the cut-off frequency. This drawback can be tackled using machine learning [33]. For example, filter banks were used to analyze the vibrations accompanying slippage [45].

These slip-induced vibrations open the gate for texture detection, as the frequencies that occur during the slippage are specific for different textures [41]. Consequently, a number of touch driven approaches were developed to complement or substitute visual perception [10]. An array of pressure sensors led to a better discriminative result [46], despite rather low bandwidth (around 50 Hz) data acquisition due to a more massive data flow.

In this work, we combined the texture classification and slip detection while using state-of-the-art deep learning (DL) techniques.

## 3. Experiments

### 3.1. Experimental Setup

**Figure 1:**
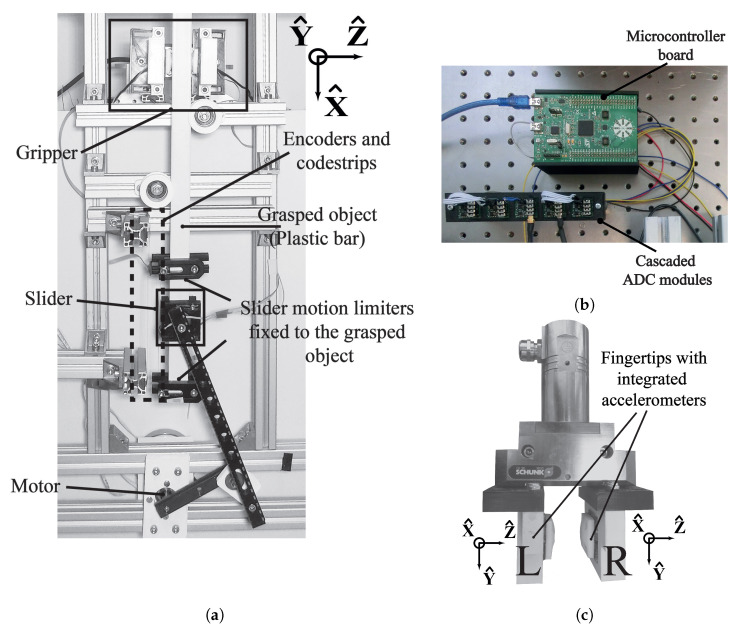
Experimental platform. (**a**) The front view of the experimental setup. (**b**) Data acquisition system consisting of microcontroller board and cascaded analog-to-digital converter (ADC) modules. (**c**) The gripper with its left (L) and right (R) fingertips with the accelerometers on the paddings.

Figure 1a depicts the front view of the setup. The setup consists of four main parts: an electric motor that generates reciprocal motion, a gripper with fingertips, encoders to measure velocity of motions, and an aluminum frame to fix all parts of the setup.

**Figure 2:**
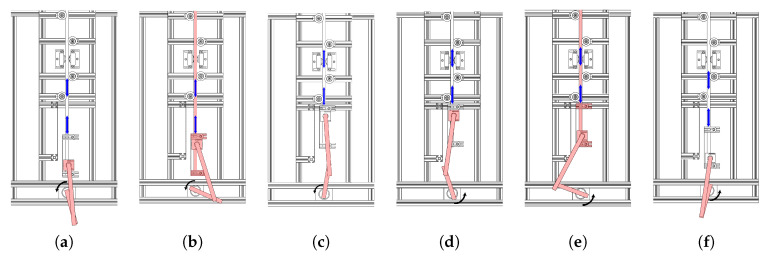
The states of the slip-slide system during one rotation of the motor. The motor is rotating counter-clockwise. Red shaded parts represent the moving objects. Blue arrows show the relative position of the grasped object in various states. The states are the following: (**a**) end of no-motion and start of sliding, (**b**) end of sliding and start of slippage, (**c**) end of slippage and start of no-motion, (**d**) end of no-motion and start of sliding, (**e**) end of sliding and start of slippage, and (**f**) end of slippage and start of sliding.

When the electric motor starts rotating, the slider moves over the grasped object until it hits a part that is rigidly connected to the grasped object. After the impact, the grasped object moves up and down. When the slider reaches the extreme points, it starts moving over the grasped object again without affecting the position of the grasped object. After a while, it hits the part fixed to the grasped object and the object starts moving again. In other words, the movement of the slider over the grasped object and movement of the grasped object alternate as the motor rotates. We define these two cases as sliding and slippage. Sliding occurs when the slider traverses across the grasped object. Slippage happens when the grasped object changes its position with respect to the gripper. In addition, we have another state when both the slider and grasped object are not moving. We call it no-motion. Figure 2 illustrates the motion transitions during one full rotation of the motor.

We used two linear encoders (AEDR-8300, 0.12 mm resolution, Broadcom Limited: San Jose, CA, USA) to measure the position of the slider and the grasped object. We computed the velocity of slippage and sliding by taking the numerical derivative of encoder readings, which, in turn, allowed us to determine the motion type.

In our setup, a gripper (Schunk, EGN100) functioned as the grasping tool (see Figure 1c). The gripper can adjust the grasping force with high precision. We made two fingertips that contain silicone rubber inserts (they isolate from the external perturbations) and installed one three-axis accelerometer (Analog Devices, ADXL335, Norwood, MA, USA) with 1.5 kHz bandwidth. We used Flex filament (Ultimaker TPU95A, Geldermalsen, The Netherlands) with 100% infill to manufacture the padding of the fingertips.

The mechanical vibrations are induced by friction. Accelerometers within the grippers capture mechanical vibrations when interacting with the external world. We used a first-order analog low-pass filter (1.5 kHz cut-off frequency) to remove high-frequency noise. In order to synchronously sample accelerometer readings, we built a data acquisition system which consists of three analog-to-digital converter (ADC) modules and a microcontroller (see Figure 1b). The ADC modules each consisting of two ADC chips (Analog Devices, AD7685) were connected in a cascaded way. An ARM microcontroller board (STMicroelectronics, STM32F3, Geneva, Switzerland) acquired data from the accelerometers at 8 kHz sampling rate in 16-bit resolution and sent them to a computer via USB as 16-bit signed integers. We also connected the linear encoders to the microcontroller.

### 3.2. Experimental Procedure

**Figure 3:**
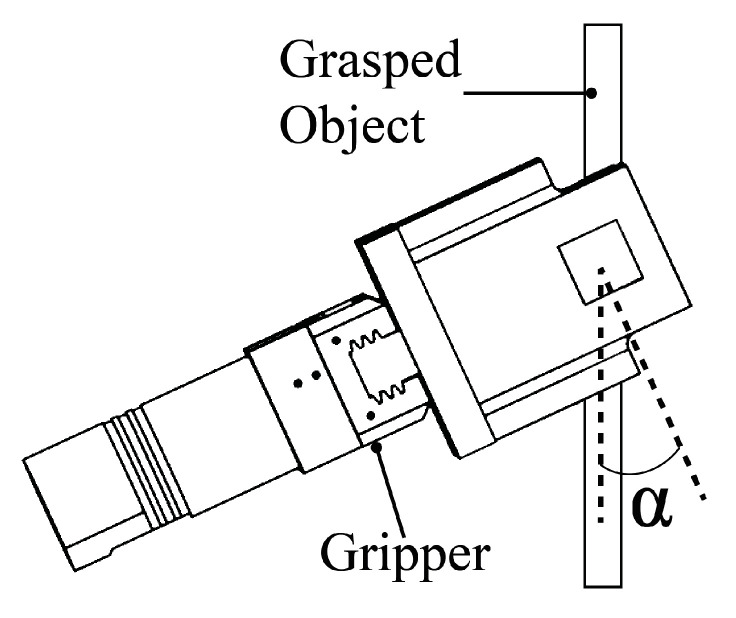
Orientation of the gripper with respect to the grasped object. The angle α was set to 0, 15, 30, and 45 degrees.

In this work, we focused on one regression (speed) and two classification (motion and texture) problems. We collected data that were related to these classification and regression tasks. For the texture classification, we obtained data from three different grasped objects (wood, plastic, and aluminum bars). In addition, we altered the angle between the grasped object and the gripper. During the experiments, this angle was set to four different values (Figure 3).

The slider was coupled with the motor through two aluminum links that were connected with a ball bearing. The rotation of the motor created slippage and sliding with varying speeds. We rotated the motor at 0.16, 0.32, and 0.48 rotations per second (rps), which generated linear motions with speed values between 0 and 0.35 m/s. A regression problem (Section 6.3) was solved due to these continuous speed values.

We conducted experiments for 36 scenarios (three objects × four orientations × three rotational speeds). We performed the following steps during the experiments: (i) Install the grasped object and orient the gripper. (ii) Trigger the data acquisition system. (iii) Rotate the motor at the desired speed. (iv) Record the data. The operation of the experimental setup and the description of its components are shown in the video submitted as supplementary material for this article.

## 4. Dataset Structure

**Figure 4:**
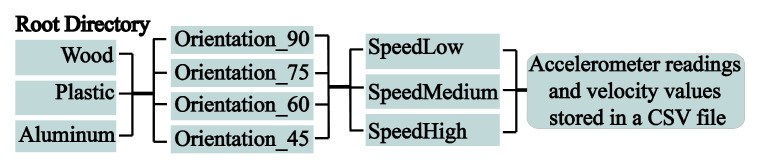
Dataset folders with hierarchical structure.

The data from each experiment were saved in a separate folder hierarchically ordered, as shown in Figure 4. The root directory contains three folders corresponding to three objects. Each of these folders has four directories corresponding to four orientations. Finally, we have three subfolders that store the data for different rotational speeds. Each folder contains a comma-separated values (csv) file with eight variables. The size of the dataset is 1.3 GB.

**Figure 5:**
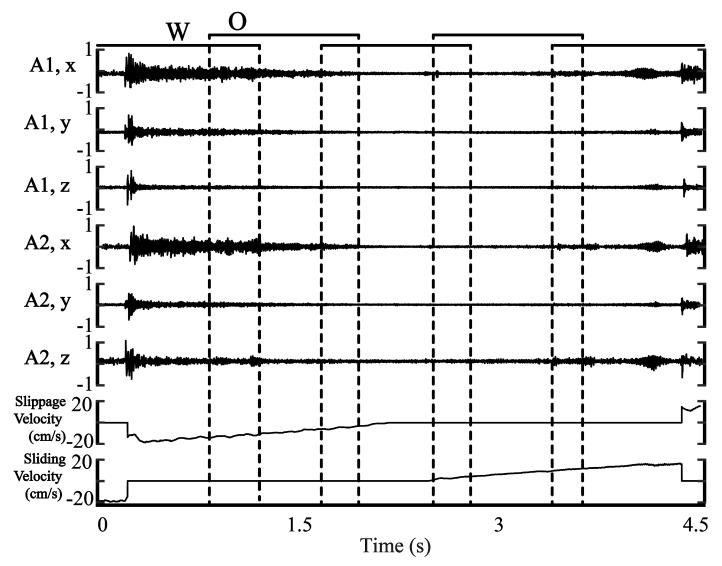
Illustration of the csv file. O-overlap, W-window size.

Each csv file contains six accelerometer measurements and velocity of slippage and sliding. After saving data to csv files, accelerometer values in the range of [−5g, 5g] were scaled to [−1, 1] range as floating point numbers. The velocity values of the motions were saved in m/s. Figure 5 illustrates a 4.5 s segment of one of these files. The last two rows represent velocity of slippage and sliding, respectively. In some cases, both velocity values are negligible when the motor is not rotating (actually, we used 5 cm/s speed threshold to define no-motion). We considered these time instances as no-motion. Accordingly, we defined three motion classes: slippage, sliding, and no-motion.

Slippage, sliding, and no-motion classes constitute 32%, 37%, and 31% of the dataset, respectively. Among texture classes, wood samples make up 40% of the dataset. The aluminum and plastic samples are 30 percent of the data each.

We used a profilometer (DektakXT, Bruker, Billerica, MA, USA) to measure the surface roughness of the objects. The measured standard deviations of the surface roughness of aluminum and plastic are 121 and 372 nm, respectively. In contrast, the standard deviation of surface roughness on wood is 6381 nm, which ensures robust grasping stability. Therefore, there were cases when both motions occurred in quick succession when conducting experiments with aluminum and plastic. This stems from the fact that they were slippery, which, in turn, allowed the grasped object to rapidly start slipping during sliding and vice versa.

The latency of slippage detection is essential for real-world operation. We tuned this parameter by varying the window size. Moreover, we used various overlapping window sizes (Figure 5). Specifically, we used the following window size as a hyperparameter: 400, 200, 100, 50, 25, and 10 samples. Correspondingly, we utilized these overlap values: 200, 100, 25, 10, and 0 samples. The window and overlap are illustrated in Figure 5. We slid over the data using these window sizes and overlapping values and generated different machine learning datasets for each combination.

We divided the data into training (80%), validation (10%), and testing (10%) sets. We preprocessed the samples before DL training. First, we removed the mean of each axis. Second, we normalized each channel to the range [−1, 1]. Inherently, slippage generates higher amplitude vibrations as compared to sliding and no-motion. We decided to normalize each sample to make DL independent from signal amplitude. This enables the detection of slippage in low speeds when the amplitude of accelerometer readings are low.

Presumably, the aforementioned classification tasks can be resolved using a small number of sensors. For instance, readings from both accelerometers might be redundant. Therefore, we tried various combinations of the accelerometers’ axes to assess these assumptions. Specifically, we had eight different settings: all six channels (L3R3), all channels of the left accelerometer (L3), x-axis of both accelerometers (LxRx), x-axis of the left accelerometer (Lx), y-axis of both accelerometers (LyRy), y-axis of the left accelerometer (Ly), z-axis of both accelerometers (LzRz), and z-axis of the left accelerometer (Lz).

## 5. Deep Learning to Decipher Vibro-Tactile Signals

In this work, we aim to detect slippage while concurrently identifying other parameters, such as texture and speed. For computational efficiency, we designed hybrid models that can tackle these tasks together.

When decreasing the window length, the number of samples increase, as shown in Table 1. For shorter window lengths, we can utilize deeper neural networks taking advantage of more samples. Therefore, we trained separate architectures for each window size and sensor combination. We applied three DL techniques: feed-forward neural networks (FNNs), recurrent neural networks (RNNs), and CNNs.

We used PyTorch library for developing DL models and conducted all model training on NVIDIA DGX-1 server.

### 5.1. FNN

**Figure 6:**
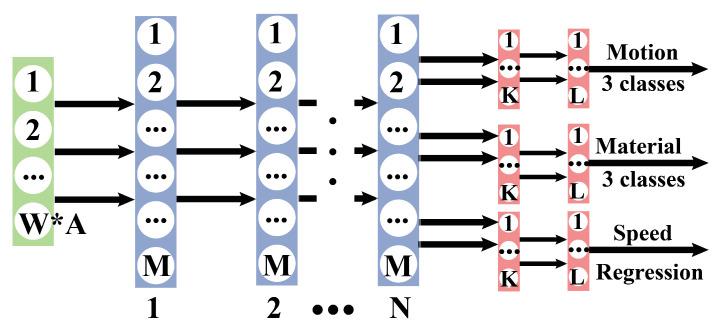
Feed-forward neural networks (FNN) network. N-number of hidden layers. W-window size. A-number of channels (1–6). M-size of hidden layers. K = 128. L = 64.

During training FNN models, we concatenated the channels into a single column vector. The classification and regression tasks shared *N* densely connected layers. *N* was varied from one to six. The number of common layer units, *M*, was varied from 128 to 4096. We utilized a grid search to identify the hyperparameters of the models. Figure 6 illustrates the topology of our FNN. We utilized two separate hidden layers for each classification and regression sub-problem.

We employed ReLU activation function, batch normalization layers in shared layers, and dropout regularization on penultimate layers (0.2). For classification (texture and motion) tasks, we used cross-entropy (CE) loss, which is defined as:

(1) $$CE=\frac{1}{n}\sum_{i=1}^{n}\left(-x\left[i\right]\left[target\right]+\log\sum_{j=1}^{m}\exp(x[i][j])\right),$$

where $x\in R^{n\times m}$, *n* is the batch size, and *m* is the number of classes.

We applied mean-square error (MSE) loss for the regression (speed) task:

(2) $$L2=\frac{1}{n}\sum_{i=1}^{n}{\left(x_{i}-y_{i}\right)}^{2},$$

where $x,y\in R^{n}$ and *n* is the batch size.

The total loss (TL) was computed, as follows:

(3) $$TL=CE_{motion}+CE_{texture}+L2_{speed}.$$

The networks were trained for 50 epochs. We used a stochastic gradient descent (SGD) optimizer with the momentum 0.9 and learning rate 0.05.

### 5.2. RNN

As our RNN topology, we used the LSTM architecture [47]. We varied the number of LSTM layers from one to three. The number of hidden layer units ranged from 256 to 1024. As in the FFN, we utilized two additional separate fully-connected (FC) layers for each task. We also used the loss defined in (3). The number of epochs was 150. We again employed SGD optimizer with the momentum 0.9. The learning rate was set to 0.05.

**Figure 7:**
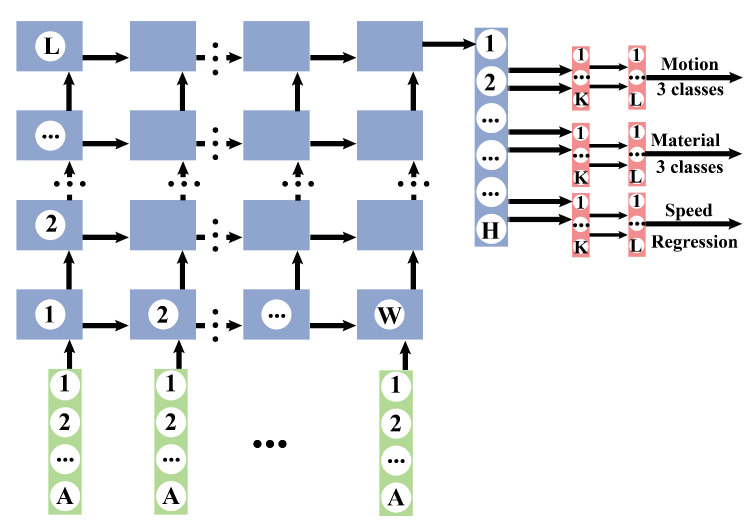
LSTM network. L-number of LSTM layers. W-window size. A-number of channels (1–6). H-hidden layer size. K = 256. L = 128.

We retrieved common features from the elements of the last hidden layer and provided them as an input to FC layers. Figure 7 depicts the architecture of our LSTM network.

### 5.3. CNN

We used AlexNet architecture as our CNN model [48]. We decreased the kernel size of the first convolutional layer to 3×3 and the number of input channels to one compared to the original version.

Before feeding our samples to the CNN model, we extracted a spectrogram of each channel to convert the original vector representation to a matrix suited for convolution operation. Subsequently, we concatenated the matrices row by row to handle multiple channels.

We slid over an input with a step half of the short-time Fourier transform (STFT) window length. Because the architecture requires a matrix input having the number of rows and columns not less than nine, we were not able to apply CNN for very short window lengths (25 and 10 for CNN).

For the window size 400, we used four different STFT window lengths: 80, 40, 20, and 10. We applied the last three options for the window size 200. STFT windows of 20 and 10 were applied for the window size 100. Finally, we used STFT window length 10 for the window size of 50.

We retrieved 4096 common features from AlexNet architecture. Subsequently, we applied two separate FC layers for each sub-task, as in FNN and LSTM networks. We utilized a SGD optimizer with the momentum 0.9 and learning rate 0.005. We used the same loss as in LSTM and FNN given in (3).

## 6. Results and Discussion

**Figure 8:**
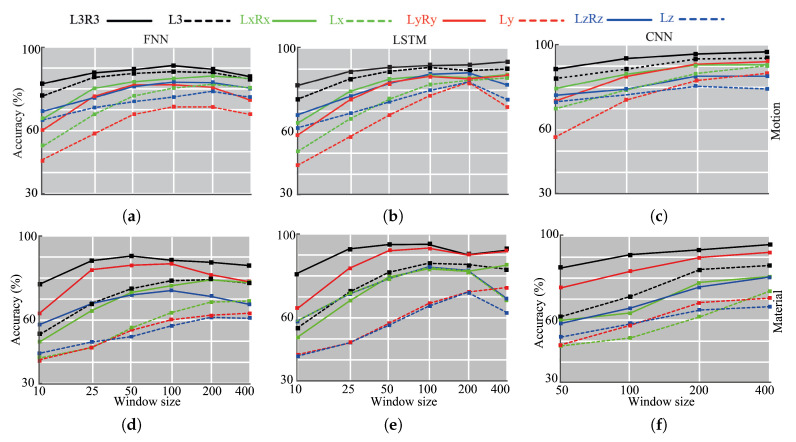
Testing accuracy values of the FNN, LSTM, and convolutional neural networks (CNN) networks for different window size values. (**a**) FNN network for motion prediction. (**b**) LSTM network for motion prediction. (**c**) CNN network for motion prediction. (**d**) FNN network for texture recognition. (**e**) LSTM network for motion prediction. (**f**) CNN network for texture recognition.

Figure 8 shows classification accuracies for different tasks and parameters. Each sub-figure incorporates eight separate lines that correspond to combinations of accelerometer readings. By observing the difference betweent rends of the same color, we see that having accelerometer readings from both sides increases the accuracy. Increasing the window size also positively affects the performance in most cases.

### 6.1. Motion Classification

The top row of the Figure 8 depicts the motion classification results of FNN, LSTM, and CNN models. We achieved 90% classification accuracy for the FNN with L3R3 configuration and the window size 100. The corresponding model had six hidden layers and 4096 units in each of them. We observed that increasing the window size improves the performance until some point. Larger windows contain more information regarding the events, however the number of samples for DL training decreases as the window size increases (see Table 1).

Compared to FNN, the LSTM network had better performance, achieving 94% accuracy. This was obtained utilizing again all six channels (L3R3) with the window size 400. The model had three layers and the number of hidden layer units was 1024. Among the three models, CNN had the highest performance, reaching 94.8% accuracy. Except for one case, we noticed positive correlations between accuracy and window length values.

Ly and Lz configurations showed the lowest performance among the eight configurations. The movement of the grasped objects was mostly aligned with x-axis, which, in turn, generated more features in this axis readings. Correspondingly, LxRx demonstrated better performance than LyRy and LzRz. The results revealed that utilizing more sensors plays an important role for the motion classification.

### 6.2. Texture Identification

The middle row of Figure 8 shows the accuracies for texture identification. The FNN model achieved 91% accuracy for the window size 50 and L3R3 configuration. The model contained seven hidden layers and 4096 units in each layer. LSTM and CNN improved the discrimination ability, further achieving 95% and 96%, correspondingly.

We noted that using y-axis of both accelerometers provided the second-best accuracy values for all window lengths. However, the y-axis of the left accelerometer did not yield high accuracy (73%). Presumably, interrelations of accelerometers provided useful features that improve accuracy.

The results of the three models demonstrated that using all axes values ensures the highest accuracy. The lowest accuracy values were mostly obtained in Ly and Lz configurations.

The dataset contained sliding, no-motion, and slippage samples when training models for the texture identification. It is questionable how the grasped object can be detected in no-motion. Indeed, installing the grasped object alters the state of the gripper. For instance, the grasping force was varied during the experiments. These changes generated different mechanical vibrations that were captured by the accelerometers. Therefore, we obtained accuracies for each motion type separately. The CNN model with L3R3 configuration and the window length 400 provided an accuracy of 99.1% for slippage, 99.3% for sliding, and 91% for no-motion. In the no-motion case, the model was able to distinguish textures but with less accuracy when compared to those of slippage and sliding.

### 6.3. Speed Estimation

The speed of the slippage was recorded in a continuous way using encoders. Hence, we tackled the slip prediction as a regression problem. The mean standard deviation (MSD) metric was used to asses the performance of the DL models. The highest average precision (MSD=3.4 cm/s) was achieved using the CNN network with the window size 400 and L3R3 configuration. For FNN and LSTM, MSD was 5.9 and 4.2 cm/s, respectively.

**Figure 9:**
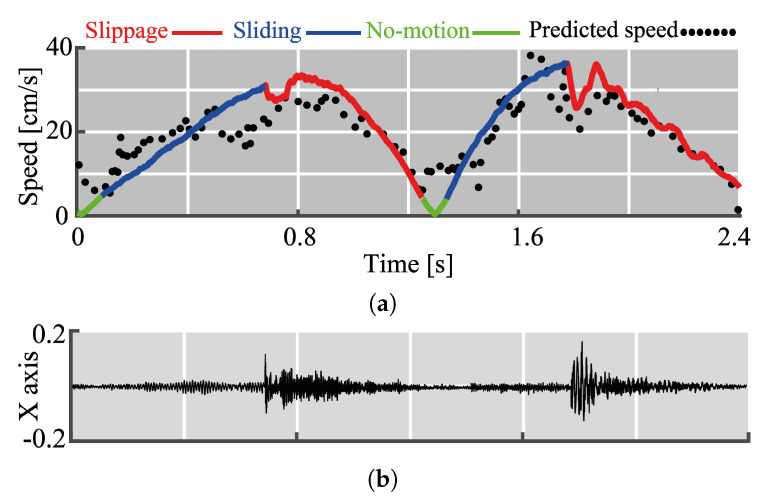
Visualization of speed estimation results. (**a**) Actual and predicted speed. (**b**) Accompanying x-axis readings of the left accelerometer.

The results of CNN model for the speed estimation are visualized in Figure 9a and vibrations accompanying these motions are shown in Figure 9b. Overall, the estimated speed followed the real one. However, there were more fluctuations during sliding. Indeed, MSD for the sliding was 1.5 times larger (4.8 cm/s) than the average one. During no-motion, MSD was smaller (2.4 cm/s) since the speed is low (the maximum speed = 5 cm/s). The MSD loss value for slippage (MSE = 3.6 cm/s) was almost twice less than for sliding. This might be due to the difference in the magnitude of the sensed vibrations during these two motions.

### 6.4. Effects of the Signal Bandwidth

DL models were trained on accelerometer readings that wer recorded at 8 kHz. The bandwidth of the accelerometer readings exceeds those of the mechanoreceptors in the human hand (1 kHz) [9]. Therefore, we hypothesized that a smaller bandwidth similar to that of human mechanoreceptors might yield similar accuracy. It would lead to a softer requirements to the system bandwidth without a significant losses in the accuracy.

**Figure 10:**
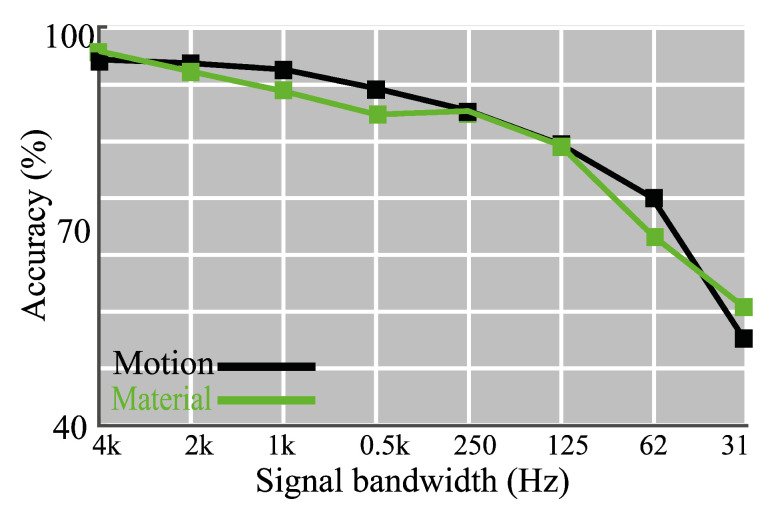
Classification results of the CNN network with L3R3 configuration and the window size 400 for different signal bandwidths.

In order to check this, we trained CNN models (L3R3, the window size is 400) for data with different bandwidth. In every step, we decreased the bandwidth by a factor of two while using a low-pass filter. We continued this process until we reached 31.25 Hz bandwidth. Figure 10 represents our results for motion and texture classification.

For the motion detection task, the accuracy was above 90% for cut-off frequencies that were above 500 Hz. The results of other classification problems also revealed a decline of the accuracy values. Motion detection accuracy abated to 80% when the cut-off frequency decreases from 500 to 125 Hz. We observed a similar decline in the texture identification task. There is a sharp drop in accuracy values for all three classification tasks when the cut-off frequency decreased from 125 to 62.5 Hz.

According to the results, it is feasible to achieve relatively high classification results for a bandwidth higher than 125 Hz. However, a bandwidth below 125 Hz is not suitable for detecting motion and classifying mechanical vibrations.

### 6.5. Computational Aspects: Latency and Memory

We computed the inference times of the DL models in a desktop computer (CPU E5-2620, 32 GB RAM, Windows 10 OS) with a GPU unit (Nvidia GTX 1080). The FNN model that provided the highest motion detection accuracy had 11 ms inference time. For the LSTM network, we obtained 80 ms inference time. The CNN model inference only took 8 ms.

However, the FNN and CNN are not preferable to LSTM with the regard of memory requirements. The size of the FNN model was 346 MB. The LSTM model required less memory when compared to FNN (86 MB). The CNN network is 240 MB.

**Figure 11:**
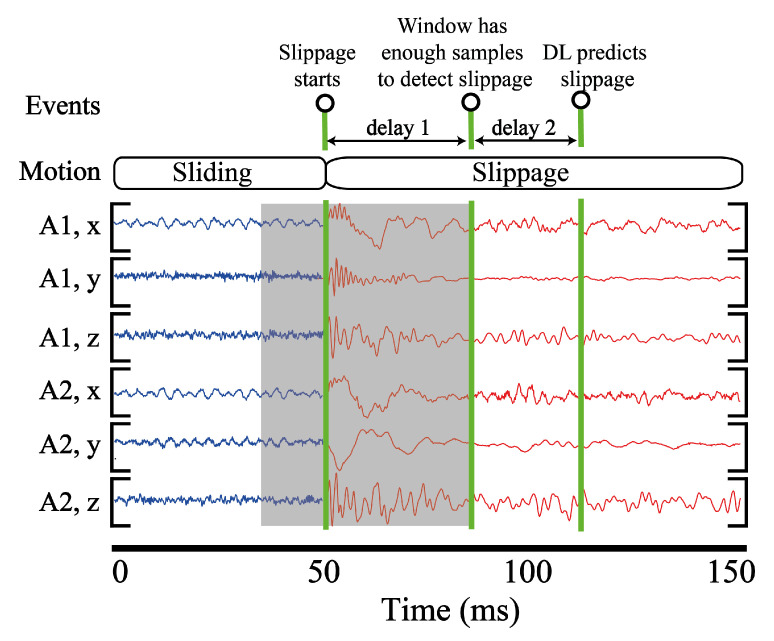
Visualization of delays in detecting slippage. Delay 1 is related to the number of slippage samples necessary for detecting slippage. Delay 2 is the inference time. Slippage starts at 50 ms. Six rows depict mechanical vibrations recorded by the accelerometers.

Figure 11 represents 150 ms time segment of accelerometer readings when slippage occurs at 50 ms. To detect this slippage event, a window should have sufficient number of slippage samples (delay 1). After that, DL models spend some time to execute computations, which is equal to the inference time (delay 2).

To compute delay in gathering slippage samples, we slid over the testing dataset with the stride 5 and window size 400. We identified transition points and instances when slippage starts detecting. Subsequently, the difference was computed between these instances. Finally, we estimated th average number of samples necessary to detect slippage. The results reveal, on average, at least 70 samples are necessary to detect slippage, which corresponds to 8.75 ms for the window size 400 and L3R3 configuration. Because the inference time is equal to around 8 ms, we estimate about 17 ms delay in detecting slippage.

## 7. Conclusions and Future Work

The results of the DL models show that vibration signals induced by friction possess essential information to identify haptic clues. Specifically, we successfully implemented FNN, LSTM, and CNN techniques to detect slippage while concurrently identifying texture and speed using high-bandwidth signals that are generated by accelerometers. Moreover, the outcomes of various combinations of accelerometer readings indicate that utilizing more sensors is integral for achieving high discrimination of motion types and textures.

The inference time and delay values in detecting slippage show that the DL models can operate in real-time applications. It facilitates dexterous manipulations of objects and rich haptic content in robot hands. Additionally, the results of the CNN network revealed that signals with bandwidth commensurate to those received by human mechanoreceptors are suitable to apply them for classifying motions and textures.

As a future work, we aim to install the gripper with fingertips on a manipulator to examine the DL models in realistic scenarios.

## Figures and Tables

**Table 1:** The number of samples for each window size.

Window size	400	200	100	50	25	10
Overlap	200	100	50	25	15	0
Number of samples	115 k	234 k	463 k	927 k	1546 k	2319 k

## Supplementary Materials

The following are available at https://www.mdpi.com/1424-8220/20/15/4121/s1, Video S1: Vibro-tactile Perception for Simultaneous Texture Identification, Slip Detection, and Speed Estimation.
